# Why do we have so many different transcripts?

**DOI:** 10.1371/journal.pbio.3003686

**Published:** 2026-03-20

**Authors:** Laurence D. Hurst

**Affiliations:** The Milner Centre for Evolution, University of Bath, Bath, United Kingdom

## Abstract

The evolutionary significance of extensive transcript diversity generated by alternative transcription initiation, splicing and polyadenylation in eukaryotes remains controversial. This Primer explores a recent study in PLOS Biology showing that the high diversity of transcripts in mammals is down to the fact that accidents happen.

## Teaser

While it is tempting to suppose that everything that happens inside our cells has a function, a recent study in *PLOS Biology* adds to the growing consensus that, for large-bodied species, the high diversity of transcripts is down to the fact that accidents happen.

Look at human RNA sequencing data and you will find that most protein-coding genes have transcripts that, compared to the most abundant transcript, use alternative polyadenylation sites, alternative transcription initiation sites, and are alternatively spliced. Indeed, for our multi-exon genes, an absence of an alternative splice form is the rare exception. Why does such diversity exist?

Perhaps, as ENCODE suppose [1], every genomic activity has a function? In evolutionary circles, this is sometimes known as Panglossian thinking [2] (also known as adaptationism, selectionism), after Voltaire’s Dr Pangloss who, lampooning Leibniz, assumed that all is for the best in this, the best of all possible worlds. But there is an alternative. Many transcripts could be simple cellular mistakes, errors. Work over the past decade has strongly tilted towards the accident/error hypothesis as an explanation for our high transcript diversity, as well as for the commonality of phenomena such as circular RNAs, RNA editing, and stop codon readthrough (for a review see [3]). A new study in *PLOS Biology* from Mi and colleagues [4] reinforces this with the largest analysis to date.

How could you know if the transcript diversity reflects accident more than selection? For any given transcript, you could ask what happens when you delete or over-express it. But if it shows an effect, does this mean it has a ‘function’? Even random transcripts can be bioactive [5]. What if it showed no effect? Could you then conclude it has no function? Perhaps you have not looked in the right conditions or cannot measure subtle effects well enough?

The trend over the past decades in evolutionary circles has been to ask a question that may seem foreign to molecular biologists: is there more diversity when species have fewer members? This needs explanation.

Possibly the most important addition to evolutionary theory in the later part of the 20th century is the so-called nearly neutral theory of evolution [6]. The nearly neutral theory is conceptually similar to the (strictly) neutral theory, seeing most differences between species at the molecular level as owing to chance changes in allele frequency, not selection. The two theories, however, make opposing predictions about rates of evolution. While the neutral theory says the rate of evolution is independent of population size, crucially the nearly neutral theory disagrees. The nearly neutral model supposes that selection will, on average, favor mutations that decrease error rates, but that, if this is weak selection, then chance changes in the frequency of such mutations (i.e., drift) can prevent selection from ‘perfecting’ the genome. Importantly, the chance effects will relatively dominate when the species concerned has fewer members, just because of the nature of chance. Thus, the nearly neutral model makes a broad prediction: you should witness more imperfection [7], more errors, in species with fewer members.

**Fig 1:**
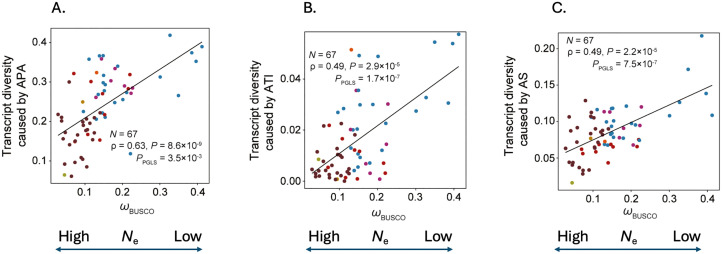
Trends in transcript diversity as a function of effective population size proxy, ω (the rate of protein evolution controlling for the background rate, employing conserved single copy genes). **A.** Transcript diversity as regards alternative polyadenylation (APA) usage. **B.** Transcript diversity as regards alternative transcript initiation (ATI) usage. **C.** Transcript diversity as regards alternative splicing (AS) frequency. Diversity is defined as the proportion of the transcriptome for each gene that is not the most abundant “canonical” form. For each figure, *N* is the sample size, *ρ* the Spearman rank correlation, *P*, the significance thereof and *P*_PGLS_, the significance of the correlation allowing for shared ancestry. Data from [4].

To be more exact, the model predicts that something called the effective population size (*N*_e_), not simply the head count of individuals, is what matters. Unfortunately, *N*_e_ is not easy to measure, and we commonly rely on proxies. Mi and colleagues [4] consider direct estimates of *N*_e_ for a limited sample of species, and three variables that should be correlated with *N*_e_ (body length, longevity, and rate of protein evolution compared to background) for more species. Using similar proxies, Bénitière and colleagues [8] recently reported that species with low *N*_e_ had a greater diversity of alternative splice forms, consistent with the nearly neutral model. Mi and colleagues [4] now extend this analysis and show that all three types of transcript diversity are higher when population sizes are smaller (Fig 1). In addition, selection should be more effective against highly expressed genes as the burden caused by misprocessing these is heavier. They find the predicted lower transcript diversity for highly expressed genes.

While this is as predicted by the ‘accidents happen when selection is inefficient’ model, surely species with small populations—such as humans recently had—are also those that are more complex. Do such species not need a greater diversity of transcripts to be more complex? One prior analysis using the number of different cell types as a measure of complexity found in favor of this model [9]. Unfortunately, they employed higher synonymous site diversity as the proxy to higher *N*_e_, which we now know is also affected by the mutation rate, and this too varies with *N*_e_. Indeed, as mutation is also a molecular error, species with a small *N*_e_ are also predicted by the nearly neutral model to have a higher rate per generation, a trend that has been observed when considering mutation rates in the span from bacteria to mammals [10]. Mi and colleagues [4] repeated the complexity analysis and find that, while transcript diversity is indeed higher in more complex species, controlling for shared ancestry and *N*_e_, cell type diversity is not significantly predictive of transcript diversity. However, for this analysis, they employed a reduced dataset as the number of cell types is not a well-resolved statistic.

With caveats then, the results add to a considerable body of evidence [3,7,8] that the relative (in)efficiency of selection provides an explanation for the between-species differences in diversity generation: diversity variation at both the transcript and mutational level is the result of accidents that selection is too inefficient to prevent, not some selection for variety.

Is that then it? Should we not bother trying to determine the (presumed) function of alternative transcripts? Some, naturally, will have a function. The theory is not about whether only one transcript from each gene is functional, just whether the overall diversity is crafted by selection for diversity or not. More particularly, as the nearly neutral model predicts, Bénitière and colleagues [8] observe that the diversity that is increased when selection is inefficient is the cloud of transcriptomically rare isoforms. These indeed look like mistakes, often having weak splice sites and premature stop codons [8]. If you want to look for an important alternative transcript, pick a relatively common one without a premature stop codon.

The paper by Mi and colleagues [4] is one in a succession of papers that, within a broader context, challenge the assumptions that led the ENCODE team to declare that the junk DNA hypothesis is dead because they found ‘activity’ at the majority of our genome [1]. Such logic frustrates the more evolutionary-minded [11], if only because it fails to even consider a null ‘accidents-happen’ model of molecular evolution. The fact that naïve DNA introduced into cells is also highly transcribed, more so than the species' own DNA, supports the same ‘accidents-happen’ model [12]. The same model provides a novel explanation for why we have so many filters in place to prevent transcripts from getting to the ribosome—the unwanted transcript hypothesis—and why these rely on features not commonly seen in ‘wanted transcripts’ [13]. But do I expect Panglossian assumptions to give way to an ‘accidents-happen’ null of human transcriptomics any day soon? Given past experience, I am not holding my breath, not least because demonstrating that a transcript truly is an error is harder than showing it has some activity, and publication bias will inevitably favor the latter over the former.
